# Analysis of context factors in compulsory and incentive strategies for improving attraction and retention of health workers in rural and remote areas: a systematic review

**DOI:** 10.1186/s12960-015-0059-6

**Published:** 2015-07-21

**Authors:** Xiaoyun Liu, Lixia Dou, Huan Zhang, Yang Sun, Beibei Yuan

**Affiliations:** China Centre for Health Development Studies, Peking University, Mailbox box 505, 38 Xueyuan Road, Haidian District, Beijing 100191 People’s Republic of China; Center for Woman and Child’s Health, China Center for Disease Control and Prevention, Beijing, China; School of Political Science and Public Administration, Wuhan University, 299 Bayi Road, Wuchang District, Wuhan China

**Keywords:** Human resources for health, Attraction, Retention, Context factors, Rural areas

## Abstract

**Background:**

Current literature systematically reports that interventions to attract and retain health workers in underserved areas need to be context specific but rarely defines what that means. In this systematic review, we try to summarize and analyse context factors influencing the implementation of interventions to attract and retain rural health workers.

**Methods:**

We searched online databases, relevant websites and reference lists of selected literature to identify studies on compulsory rural service programmes and financial incentives. Forty studies were selected. Information regarding context factors at macro, meso and micro levels was extracted and synthesized.

**Results:**

Macro-level context factors include political, economic and social factors. Meso-level factors include health system factors such as maldistribution of health workers, growing private sector, decentralization and health financing. Micro-level factors refer to the policy implementation process including funding sources, administrative agency, legislation process, monitoring and evaluation.

**Conclusions:**

Macro-, meso- and micro-level context factors can play different roles in agenda setting, policy formulation and implementation of health interventions to attract and retain rural health workers. These factors should be systematically considered in the different stages of policy process and evaluation.

## Introduction

There is a worldwide issue that remote and rural areas tend to have far less human resources for health (HRH) than their population needs [1]. Universal health coverage will be impossible to achieve if there remain populations with insufficient access to qualified health workers [2]. Many countries have designed and implemented interventions to attract and retain health workers in remote and rural areas [3]. Most of the interventions are from high-income countries, such as the Medical Rural Bonded Scholarship (MRBS) Scheme as an Australian Government recent initiative designed to address the doctor shortage outside metropolitan areas across Australia [4]. A report from the World Health Organization (WHO) summarized and categorized these interventions into four broad categories: education, regulations, financial incentives and personal and professional support [5]. However, currently available evidence is contradictory and draws a complex picture on the intervention effectiveness. For example, one systematic review on financial incentives in underserved areas identified nine studies with increased uptake of rural jobs, two with negative findings and two with no significant differences [6].

These interventions were developed and implemented in various contexts and with different processes, which might help explain the variations of intervention effectiveness. The literature systematically reports that interventions to attract and retain health workers in underserved areas need to be context specific but rarely defines what that means [5,7]. A systematic review without consideration of these context factors may have limited value in providing guidance for countries to develop their own strategies.

Policy context includes all environmental factors under which a policy is made and implemented. Context factors in health policies can be classified in various ways according to the nature of the factors or the role they play in policymaking process. Leichter categorized context factors into situational factors, structural factors, cultural factors and environmental factors [8]. Collins discussed the context factors of health system reform in six dimensions: demographic and epidemiological change, processes of social and economic change, economic and financial policy, politics and the political regime, ideology, public policy and the public sector, and external factors [9]. Implementation process can also be considered as one important aspect of context [10]. While policy processes are usually divided into different stages including agenda setting, policy formulation and policy implementation, these context factors may have different influences on these policy stages.

Therefore, the objective of this review is to identify key context factors that policymakers should consider when they design and implement interventions. The context factors are discussed at three levels. Macro-level context factors include political, economic and social factors. Meso-level context factors include health system factors. Micro-level context factors refer to the implementation process of the intervention. This categorization will help analyse the potential roles that these context factors may play in the different stages of the policy process. We hope this analysis can help policymakers properly interpret the mixed evidence from existing literature and therefore help countries to design and implement context-specific intervention strategies to attract and retain rural health workers.

## Methods

### Search strategies

#### Data sources

Literature from three different sources was searched: online databases, relevant websites and reference lists of selected literature.

Online databases included PubMed, EMBASE, PsycINFO, The Cochrane Central Register of Controlled Trials (CENTRAL), ERIC, JSTOR, EconLit, SSRN, IDEAS, System for Information on Grey Literature in Europe (OpenSIGLE), National Technical Information Service (NTIS), ProQuest Dissertation & Theses Database, ISI Proceedings and Popline.

#### Search terms

Four types of terms were applied in the literature search: 1) terms about study settings: remote, rural, primary health care, underdevelopment, under-served areas; 2) terms about participants: health personnel, health manpower, health professional, physicians, nurses; 3) terms about interventions: physician incentive plans, compulsory service, motivation, training; and 4) terms about effectiveness: personnel turnover, attraction, retention, recruitment.

Six websites were searched including the WHO website, The Global Health Workforce Alliance, World Bank, Capacity Project website, Asia Pacific Action Alliance on Human Resources for Health and Google Scholar (first 50 pages).

In the literature search process, no limitation was specified on the publication dates which meant literatures published in all time on this topic were eligible for the literature search.

### Study selection

**Figure 1:**
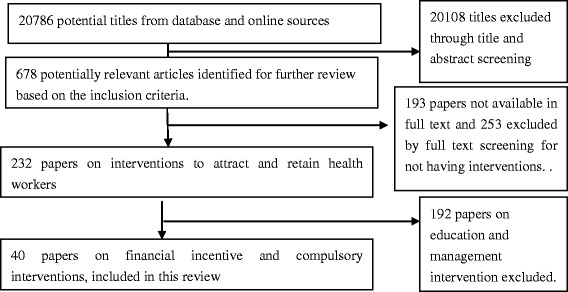
The selection process for studies included in the review.

The review focuses on two types of intervention strategies: compulsory rural services programmes and direct and indirect financial incentives. The target population may include both existing health professionals and medical students. Studies were included in the analysis when context or process information of the interventions was discussed. Literatures were excluded if the papers had no introduction of interventions or if interventions were not about compulsory rural service programmes and financial incentives. Two reviewers independently assessed potential studies for inclusion and resolved disagreements through discussion. The study selection process is shown in Figure 1.

### Data collection and analysis

A standard data extraction form was used to extract data on study characteristics (title, author, year and study methods), intervention design, effectiveness and context factors. Information on context issues and process were coded and grouped into three levels: 1) macro-level context factors include political, economic and social factors; 2) meso-level context factors include health system factors; and 3) micro-level context factors refer to the implementation process.

We assessed the methodological quality of the included studies with the criteria developed by Hawker and colleagues [11,12]. We used eight dimensions for quality assessment. For each dimension, we rated the quality of included studies from 1 (good) to 4 (very poor). The overall methodological quality for each study was calculated using the average score of the eight dimensions. Then the quality of included studies was grouped into four levels (1.00–1.49 = good; 1.50–2.49 = fair; 2.50–3.49 = poor; 3.50–4.00 = very poor) [12].

In total, 40 studies were included in this review, as shown in Table 1 [1,6,13-50]. Twenty-five studies were rated “good” in methodological quality, 11 “fair”, 3 “poor” and 1 “very poor”. The context factors are reported in Table 2.

**Table 1:** **Included studies**

	**First author’s last name**	**Year of publication**	**Country involved**	**Study design**
1	Dambisya [1]	2007	Angola, Botswana, DRC, Kenya, Lesotho, Madagascar, Malawi, Mauritius, Mozambique, Namibia, South Africa, Swaziland, Tanzania, Uganda, Zambia, Zimbabwe	Descriptive study
2	Bärnighausen [6]	2009	sub-Saharan Africa	Cost–benefit analysis
3	Adjei [13]	2009	Ghana	Descriptive study
4	Baumann [14]	2006	Uganda, Thailand, United Kingdom and Canada	Descriptive study
5	Bhattacharyya [15]	2001	Nigeria, Ethiopia, Bangladesh, Zambia, Guatemala	Descriptive study
6	Caffrey [16]	2006	Malawi	Descriptive study
7	Cavender [17]	1998	Ecuador	Cross-sectional study
8	Chomitz [18]	1998	Indonesia	Retrospective cohort study
9	De Arellano [19]	1981	USA	Descriptive study
10	Ditlopo [20]	2011	South Africa	Qualitative study
11	Duttera [21]	2000	USA	Retrospective cohort study
12	Efendi [22]	2012	Indonesia	Descriptive study
13	George [23]	2013	South Africa	Cross-sectional study
14	Koot [24]	2005	Zambian	Qualitative study
15	Laven [25]	2005	Australia	Case–control study
16	Matsumoto [26]	2008a	Japan	Prospective cohort study
17	Matsumoto [27]	2008b	Japan	Prospective cohort study
18	Matsumoto [28]	2009	Japan	Prospective Cohort study
19	Matsumoto [29]	2010a	Japan	Prospective cohort study
20	Matsumoto [30]	2010b	Japan	Retrospective cohort study
21	Meliala [31]	2013	Indonesia	Descriptive study
22	Pagaiya [32]	2009	Thailand	Descriptive study
23	Pathman [33]	2000a	USA	Cross-sectional study
24	Pathman [34]	2000b	USA	Descriptive study
25	Pathman [35]	2004	USA	Cross-sectional study
26	Peña [36]	2010	Chile	Descriptive study
27	Reid [37]	2004	South Africa	Retrospective cohort study
28	Renner [38]	2010	USA	Retrospective cohort study
29	Ross [39]	2004	South Africa	Descriptive study
30	Ross [40]	2007	South Africa	Qualitative study
31	Shroff [41]	2013	India	Qualitative study
32	Thaker [42]	2008	USA	Descriptive study
33	Weiss [43]	1980	USA	Descriptive study
34	Wibulpolprasert [44]	2003	Thailand	Descriptive study
35	Wiwanitki [45]	2011	Thailand	Descriptive study
36	Wongwatcharapaiboon [46]	1999	Thailand	Retrospective cohort Study
37	Yumkella [47]	2009	Kenya, Ghana, Swaziland, Zambia, Uganda,	Descriptive study
38	Zurn [48]	2010	Senegal	Descriptive study
39	Gow [49]	2013	Zambia	Cross-sectional study
40	Goma [50]	2014	Zambia	Cross-sectional study

**Table 2:** **Categorization of context factors in the review**

**Context categories**	**Context factors**	**Descriptions of the context factors**	**Number of selected studies reporting the factors**
Macro level	Political factors	Democratic election	1
		Post-conflict	2
		Social movement towards equity	3
	Economic factors	Fiscal capacity of a country or organization	8
		Rising cost of medical education	7
	Social factors	Traditional culture and ethics	7
Meso level	Health system	Deficit and maldistribution of health workforce	34
		Private services	10
		Decentralization	6
		Health financing	5
Micro level	Implementation process	Monitoring and evaluation	10
		Consultation and engagement of actors	26
		Funding sources	22
		Legislation process	7

## Results

### Study characteristics

Fifteen studies were from high-income countries, 20 from low- and middle-income countries and 5 from a mixture of both. Eighteen studies were descriptive studies (no design study, just a plain description of the interventions, context factors, implementation process or effectiveness). Other study designs included cohort study (10), cross-sectional survey (6), qualitative study (4), case–control study (1) and cost–benefit analysis (1).

### Interventions and effectiveness

In the selected studies, there were four broad types of xfinancial incentives: scholarship, loan, loan repayment and direct financial incentives. These financial incentive and compulsory rural service programmes were usually combined together. Effectiveness of these interventions was mixed. Studies in Japan and USA showed that financial incentives (scholarship and loan repayment) together with compulsory rural service programmes were successful in improving the attraction and retention of rural health workers [26-36,33-35], while studies in Zambia showed that the health worker retention scheme did not meet its policy objectives to address the shortage of rural health workers [49,50].

### Macro-level context factors

#### Political factors

Six out of 40 studies reported political factors relevant to the interventions (Table 2). A post-conflict situation after civil war usually meant fragile health systems and shortfall of resources including HRH [1]. The timing of introducing a rural allowance policy to address geographical inequities in health personnel distribution in South Africa right before its second democratic election in 2004 suggested a political motive [20]. Social movement towards equity in South Africa after the abolishment of apartheid [23] and in Thailand [46] draws more attention to the HRH disparity between urban and rural areas.

#### Economic factors

Fifteen out of 40 studies reported economic factors. Interventions, especially the financial incentives, require financial commitments. The fiscal capacity of a country or organization may largely affect not only its enthusiasm to address HRH problems but also its actual choices. In high-income countries such as Australia and Chile, compensation for tuition fees can be from 5000 to 10 000 US dollars per year [25,36], while in many African countries, a popular intervention was to provide moderate lunch or transportation allowance [47].

Another economic-related factor is the rising cost of medical education. This is particularly a concern in the USA where the tuition fees for medical students are extremely high. More than 80% of medical students carried educational loans after graduation. Looming training debt may force some young physicians into the higher paying, non-primary care specialties and thereby undermining national efforts to expand the primary care workforce. In this context, the loan repayment programme became increasingly popular to young medical graduates with training debt. The programme pays for their loan on a regular basis while in return the medical graduates provide service in rural areas. It was reported that one quarter of medical graduates committed to this support-for-service programme [33]. As the medical education is gradually opened to the private sector and consequently the escalating cost for medical training that is happening in many countries such as Thailand [45], loan and loan repayment programmes may become a suitable intervention to attract and retain rural health workers.

#### Social factors

Seven out of 40 studies reported social factors. Social culture and ethics are other factors to be considered in intervention design. In some Asian countries, there is a cultural reluctance to borrow money from outside the circle of family relatives [51]. This may make a loan repayment programme infeasible in that context. In Ecuador and Bolivia, there was a social belief that health professionals should compensate for the free medical education they received by serving in the rural areas [17]. Helping the local community after graduation was also the motivation for some South African medical students to choose a rural job [40]. However, forcing young physicians into rural work can demoralize them. Controversy concerns were raised about whether mandatory service was ethically acceptable [45].

### Meso-level context factors

#### Deficit and maldistribution of health workforce

Deficit and maldistribution of health workforce are the essential background factors which initiate the whole idea of attraction and retention. Thirty-four out of 40 studies reported health workforce maldistribution. For example, in South Africa, there was big gap between urban and rural areas due to the internal migration of health workers [23]. In Indonesia, only 20% of doctors are located in rural areas, serving 70% of the population [31]. As a consequent, both countries introduced interventions including rural allowances and other financial incentives to address the HRH problems.

#### Private health services

Ten out of 40 studies reported context factors related to private health services. Many countries have a long history of public services. In Thailand, a unique public service system assured that all medical graduates worked as government employees to provide medical care at public health facilities. In 1968, the Thai government set high medical education fees for public medical schools and launched a programme of mandatory rural service which required all medical graduates to work at public medical facilities for at least 3 years in exchange for a waiver of tuition fee [32,44].

Although public service is still the mainstream choice in some countries, things have changed in the past decades. Increasing number of private medical schools and the promotion of private health care enforced competition for certain medical specialties. In Thailand, the number of new private hospitals had a 3.3-fold increase in 10 years from 1000 in 1985 to 3300 in 1995 [45]. These private hospitals drained physicians from the mandatory rural public service system.

#### Decentralization of health system

Decentralization of the health system can be defined as the transfer of authority, or disposal of power, in health planning, management and decision-making from a higher to lower level of government [52]. Six out of 40 studies reported context factors related to decentralization which can provide states/districts with wider choice and greater flexibility to better meet the needs of local communities. In Zambia, a decentralization policy was reported to drastically change the labour relations in the health sector [24]. Senegal decentralized public schools for more local training and recruitment of health workers [48].

#### Health financing

Five out of 40 studies reported health financing as another health system factor. Thailand introduced universal health coverage in 2001 and introduced capitation-based payment reform for outpatient services. This created a strong incentive for more equitable distribution of HRH [44]. Introduction of user fees in Uganda in the 1990s motivated doctors to jobs with high pay and better working conditions [1].

### Micro-level factors

#### Monitoring and evaluation (M&E)

Monitoring and evaluation (M&E) help monitor the implementation progress, identify and address emerging problems and track the intervention outputs and outcomes. Ten out of 40 studies introduced their efforts on M&E. There was often lack of information on how well the interventions were implemented [41]. Sometimes, these programmes even tended not to formally evaluate themselves or document their successes, as they often lacked the funds, expertise and mandate to do so [33].

#### Consultation and engagement of actors

There is no clear picture of how different actors were consulted and involved in the policy process. Twenty-six out of 40 studies reported this factor. Although, in some countries, it was reported that wide consultations were conducted during the policy process [24], in most cases, the process was considered weak and uncoordinated [20]. In Chile, trade unions emerged as strong lobby groups in the policy process [36].

Government bodies including health- or education-related authorities were commonly responsible for implementing the interventions. In a USA study, most programmes were administered by state education and finance authority. Other uncommon administration organizations included non-profit corporations, medical or nursing schools [35].

#### Funding sources

Twenty-two out of the 40 studies identified sources of intervention funding. In high-income countries, one key issue of funding is the balance between central and local governments. Pathman et al. reported that most programmes (*n* = 13) in the USA were funded by state legislatures using general tax revenues; 4 were entirely federally funded [35]. In Japan, Jichi Medical University had run a model programme with the combination of scholarship and compulsory rural service since 1972. This programme was equally funded by 47 prefecture governments in Japan [27].

Many low-income countries had to rely on international funding to support their intervention programmes. International funding may provide opportunity for the low-income countries to afford the costly interventions, although not all donors are willing to do so.

There were some additional funding sources, including buyout funds from earlier participants, private non-profit organizations [35] or even tax levies from nursing licence applications [42].

#### Legislation process of intervention strategies

Seven out of 40 studies reported legislation process in the policy development and implementation process. Some countries implemented the financial incentives and compulsory rural service programmes in the format of law. Most states in the USA had such a law. In Chile, one of the landmarks in the implementation of the Rural Practitioner Programme was the enactment of Law 15076 in 1963 which was reformed into Law 19664 in the year 2000 [36]. The main interventions in the Rural Practitioner Programme were a paid residency in a university hospital plus attractive salaries and benefits. The law granted autonomy from political support and ensured sustainability of the programme over time. It also established a secure financing mechanism.

## Discussion

Although context factors are widely considered important in the literature, these factors are rarely reported and analysed systematically. The context factors presented in this review are derived only from the available literature which may not necessarily cover all relevant context factors, due to lack of research in this specific area. For example, limited evidence was found to discuss the role of universal health coverage policy and health worker attraction and retention [53].

Although the selected studies in this review reported different context factors, there is very limited information in the original studies analysing whether or not these context factors have positive or negative influence on the development and implementation of the strategies. The review tries to discuss the potential influence of different context factors on the various policy stages.

Policy analysts usually tend to break down health policy process into a series of stages though acknowledging this does not necessarily reflect the exact process in the real world [54]. This theoretical model usually consists of agenda setting, policy formulation and policy implementation. The context factors identified in this review may have certain influence on different stages of the policy cycle, as discussed below.

Meso-level factors can play a critical role during the agenda-setting stage. In the area of attraction and retention of health workers, analysing the situation of health workforce distribution between different regions should be the first step of policy analysis. Other health system factors should also be considered when interpreting the maldistribution of health workers between. For example, the growing private sector is one of the forces attracting health workers to urban area [55]. A decentralized health system may promote more dynamic flows of health workers in the labour market, in which case the rural areas are in a disadvantaged position to attract and retain their health workers due to their disadvantages in working and living conditions [56].

Macro-level factors should be carefully considered during the policy formulation stage. In different political systems, the governing body may have special preference for financial incentive interventions or compulsory regulations in order to address the deficit of health workers in rural areas. The choice of intervention strategies will largely depend on the economic development and financial capacity of the central or local government. Policy formulation process should also carefully consider the social acceptance of potential interventions, according to their specific social culture and values.

Micro-level factors that are essential in policy implementation and evaluation stage usually do not receive sufficient attention [57]. Stakeholders are not always properly consulted and involved in the policy implementation process. M&E are no doubt one of the most important parts of the intervention programme. Without M&E, one cannot tell how the intervention is implemented, cannot solve emerging problems during the implementation and cannot track the outputs and outcomes of the interventions. However, most of the interventions are not rigorously monitored and evaluated. Furthermore, funding sources may not be sustainable to implement these policies.

The relationships described above regarding the context factors and different stages of policy process are not exhaustive. There might be other direct and indirect relationships between the context factors and the policy stages. For example, in evaluating the effectiveness of interventions, one may also need to consider the macro-level factors (political and economic factors) and meso-level factors (health system factors) in interpreting why some intervention strategies work well in this country context but not in other settings.

Macro-, meso- and micro-level context factors should be carefully considered when formulating, implementing and evaluating strategies to attract and retain health workers in rural areas. This review may help low- and middle-income countries to properly adopt WHO-recommended strategies [5]. First, they need to analyse their specific health system to assess the distribution of health professionals and investigate the root causes in health system. While adopting internationally proven intervention strategies, local social, economic and political factors should be checked to ensure applicability and transferability [58]. Last but not least, a carefully designed implementation and evaluation plan is crucial for the success of any interventions to attract and retain health workers to rural and remote areas.
